# Expanding the Clinical Utility of Targeted RNA Sequencing Panels beyond Gene Fusions to Complex, Intragenic Structural Rearrangements

**DOI:** 10.3390/cancers15174394

**Published:** 2023-09-02

**Authors:** Kathleen M. Schieffer, Amanda Moccia, Brianna A. Bucknor, Eileen Stonerock, Vijayakumar Jayaraman, Heather Jenkins, Aimee McKinney, Selene C. Koo, Mariam T. Mathew, Elaine R. Mardis, Kristy Lee, Shalini C. Reshmi, Catherine E. Cottrell

**Affiliations:** 1 The Steve and Cindy Rasmussen Institute for Genomic Medicine, Nationwide Children’s Hospital, Columbus, OH 43215, USA; 2 Department of Pathology, The Ohio State University, Columbus, OH 43210, USA; 3 Department of Pediatrics, The Ohio State University, Columbus, OH 43210, USA; 4 Department of Pathology and Laboratory Medicine, Nationwide Children’s Hospital, Columbus, OH 43205, USA

**Keywords:** gene fusion, structural rearrangement, intragenic, cancer, hematologic, solid tumor

## Abstract

**Simple Summary:**

In blood cancers and solid tumors, genetic changes serve to initiate and promote cancer. These genetic changes can include rearrangements to genes which can alter gene function. Identification of gene rearrangements through molecular laboratory tests may help guide clinical care in patients with cancer. Different types of gene rearrangements can occur, including those within a gene and those between genes. Rearrangements occurring within a gene can be difficult to identify using current computational approaches. Our clinical laboratory designed sequencing panels for blood cancers and solid tumors to detect rearrangements within and between genes. In this study, we discuss our three-year experience using a laboratory method of targeted sequencing to detect gene rearrangements. We highlight our approach and the clinical utility for the reporting of rearrangements both within and between genes.

**Abstract:**

Gene fusions are a form of structural rearrangement well established as driver events in pediatric and adult cancers. The identification of such events holds clinical significance in the refinement, prognostication, and provision of treatment in cancer. Structural rearrangements also extend beyond fusions to include intragenic rearrangements, such as internal tandem duplications (ITDs) or exon-level deletions. These intragenic events have been increasingly implicated as cancer-promoting events. However, the detection of intragenic rearrangements may be challenging to resolve bioinformatically with short-read sequencing technologies and therefore may not be routinely assessed in panel-based testing. Within an academic clinical laboratory, over three years, a total of 608 disease-involved samples (522 hematologic malignancy, 86 solid tumors) underwent clinical testing using Anchored Multiplex PCR (AMP)-based RNA sequencing. Hematologic malignancies were evaluated using a custom Pan-Heme 154 gene panel, while solid tumors were assessed using a custom Pan-Solid 115 gene panel. Gene fusions, ITDs, and intragenic deletions were assessed for diagnostic, prognostic, or therapeutic significance. When considering gene fusions alone, we report an overall diagnostic yield of 36% (37% hematologic malignancy, 41% solid tumors). When including intragenic structural rearrangements, the overall diagnostic yield increased to 48% (48% hematologic malignancy, 45% solid tumor). We demonstrate the clinical utility of reporting structural rearrangements, including gene fusions and intragenic structural rearrangements, using an AMP-based RNA sequencing panel.

## 1. Introduction

Hematologic and solid malignancies develop as the result of acquired somatic alterations or chromosomal rearrangements that promote aberrant activation of oncogenic signaling pathways or inactivation of tumor suppressor genes. Structural rearrangements, including gene fusions and intragenic deletions or duplications, are becoming increasingly recognized as drivers of pediatric and adult tumors (benign and malignant). It is estimated that >20% of human cancers are associated with a gene fusion event [1]. In addition, complex structural rearrangements are becoming increasingly recognized as driving events in cancer [2,3,4,5,6,7,8,9,10,11,12,13,14,15]. While fusions result from balanced or unbalanced structural rearrangements [16], the biological mechanisms underlying intragenic duplications or deletions are varied and gene dependent. An internal tandem duplication (ITD) results from an in-frame duplication of variable length that alters gene function.

ITDs have clinical significance in numerous cancer types. For example, an ITD occurring predominantly in exon 14 of the receptor tyrosine kinase, *FLT3*, is a well-established prognostic marker for acute myeloid leukemia [12,17]. The *FLT3* in-frame tandem duplication encodes for a fragment of the juxtamembrane domain, a regulatory domain involved in maintaining an inactive conformation in the absence of ligand [18] resulting in ligand-independent dimerization and phosphorylation to promote constitutive signaling. Furthermore, ITDs in the growth factor receptors *EGFR* and *FGFR1* are characteristic driver events described in pediatric and adult solid and central nervous system (CNS) tumors. The *EGFR* and *FGFR1* intragenic structural rearrangements result in the tandem duplication of the entire tyrosine kinase domain, promoting protein autophosphorylation and constitutive receptor activation [2,5,6,8,10,13,14,15,19,20]. Notably, in the setting of both *FLT3* and *EGFR* ITDs, targeted therapies have demonstrated clinical utility [4,6,12,13]. Intragenic deletions occur when a portion of genomic material within a gene is lost. Dependent on the gene and the deletion event, mechanistically these can result in the inactivation or altered function of the translated protein product. A classic example in pediatric acute lymphoblastic leukemia involves the *IKZF1* gene, whereby variably deleted exons generate multiple alternatively spliced transcript isoforms [11]. The presence of this event helps guide patient risk stratification [21,22].

Identification of structural rearrangements may occur by both cytogenetic and molecular methodologies, including conventional karyotype, fluorescence in situ hybridization (FISH), reverse transcription PCR (RT-PCR), microarray analysis, and targeted next generation sequencing (NGS)-based assays [16,23,24,25]. Each methodology has inherent advantages and disadvantages that need to be taken into consideration during clinical interpretation (Table 1). For example, conventional karyotype analysis relies on the ability to culture disease-involved cells, and resolution is limited to what is detectable by microscopic analysis of chromosome morphology and banding patterns. FISH may provide a rapid turnaround time and information about numerical and structural abnormalities; however, data are limited to that observable in interphase or metaphase studies. Atypical and novel signal patterns, in particular, can be challenging to interpret relative to the predicted downstream impact on the gene products. Targeted RT-PCR is rapid and cost-effective for interrogating a limited number of well-characterized structural rearrangements at the level of the transcript; however, atypical breakpoints or poor-quality tissue (e.g., highly fragmented or cross-linked nucleic acid derived from some formalin-fixed paraffin-embedded (FFPE) samples) may hinder assay performance and result interpretation. NGS methods have high throughput capability to detect structural rearrangements by either a targeted or unbiased approach at the transcript level. Although the cost of sequencing has decreased over time, this approach may still be cost-prohibitive in some clinical settings and requires a strong bioinformatics infrastructure. In addition, identification of certain alterations, such as ITDs from paired-end short-read sequencing data, can still prove to be bioinformatically challenging. Bioinformatic tools such as CICERO [26] and arriba [27] may aid in an unbiased detection of ITDs and gene fusions in RNA-sequencing data, while tools such as Pindel [28] (small to moderate size insertions/deletions) may be useful for DNA-based NGS technologies.

Herein, we describe a three-year experience with an anchored multiplex PCR (AMP)-based assay to generate targeted RNA sequencing libraries for the Illumina platform. Additionally, we highlight the utility of AMP-based targeted RNA-sequencing to identify complex intragenic structural rearrangements, including internal tandem duplications (ITDs) and intragenic deletions in tumors from individuals with solid cancers and hematologic malignancies.

## 2. Materials and Methods

### 2.1. Study Participants

This retrospective study evaluated tumors and disease-involved samples (*n* = 608) from individuals undergoing diagnostic testing for targeted gene fusion analysis on the basis of external and internal clinical orders received by The Steve and Cindy Rasmussen Institute for Genomic Medicine Clinical Laboratory at Nationwide Children’s Hospital from 1 January 2019 through 31 December 2021. Testing was performed using a gene panel specific for either hematologic malignancy (FusionPlex Pan-Heme (Part #DB0247; FusionPlex Pan-Heme EPOR add v1.2; ArcherDx, Boulder, CO, USA) or solid tumors (FusionPlex Pan-Solid (Part #DB0177; FusionPlex NWC Pan Solid Tumor v1.0; ArcherDx, Boulder, CO, USA), depending on the indication for study. The Pan-Heme panel was offered throughout the entirety of the three-year timeframe, while the Pan-Solid panel was clinically offered starting 1 April 2019. Demographic information, including sex, age, specimen source, and indication for testing, was collected from the requisition form. This study was approved by the Institutional Review Board at Nationwide Children’s Hospital.

### 2.2. Library Preparation and Sequencing

This assay evaluates 154 genes for Pan-Heme and 115 genes for Pan-Solid, in addition to four control genes (Table S1). Disease-involved specimens, including peripheral blood, bone marrow, pleural fluid, and lymph node tissue for hematologic analysis, and snap frozen or FFPE for solid tumor analysis, underwent internal pathology review to assess for adequate disease involvement (>10% disease content for disease-involved peripheral blood/bone marrow/pleural fluid or snap frozen tissue, and >25% for FFPE tissue). Custom AMP-based targeted RNA sequencing assays comprising separate panels for hematologic and solid tumors were designed, with the methodology utilizing a unidirectional gene-specific primer (GSP2) which targets specific exons as part of a designed panel to enable identification of both known (recurrently described) and novel gene fusions and isoforms (see Table S1 for gene content and GSP2 primer coordinates). GSP2s were designed to cover the common structural rearrangement breakpoints and may not target all exons in the gene. Due to the unidirectional nature of the GSP2s, PCR products can amplify across unknown breakpoints, allowing for the identification of novel gene fusions and intragenic structural rearrangements. Single nucleotide variation, small intraexon insertions/deletions (with the exception of *BCOR* ITD and *FLT3* ITD, both of which have been curated within the Archer database), and whole gene duplications/deletions cannot be detected by this assay.

### 2.3. Sequencing

Libraries were generated following the manufacturer’s recommendations, in addition to performing quantitative PCR (qPCR) amplification for sample quality. If the qPCR value was below 30 CT, the sample quality was deemed sufficient to continue to library generation. This assay utilizes unidirectional GSP2 to amplify molecular barcodes ligated onto cDNA fragment ends. Amplified products were sequenced using the MiSeq (Illumina, San Diego, CA, USA) sequencer. Each FusionPlex library preparation batch included a control sample comprising a 10% dilution of the SeraSeq Fusion RNA mix v3 control (Item No. 0710-0431, SeraCare Life Sciences Inc., Milford, MA, USA), from which a library was generated to assess for batch and run-level quality and accuracy in sensitively calling fusions and structural rearrangements.

### 2.4. Sequence Data Analysis

BCL conversion was performed in an Amazon Web Services (AWS) cloud process using bcl2fastq v2.20.0.422 (Illumina, San Diego, CA, USA). The resulting FASTQ file was transferred to the Archer Analysis Software v6.2.7 virtual instance hosted internally. Samples originally processed using Archer Analysis Software v5.1.3 were reprocessed through v6.2.7. The data analysis was configured with default settings using a gene transfer file (GTF) specific to the utilized gene panel (Pan-Solid (dSA09260 Fusion Plex NWC Pan Solid Tumor v1.0, Table S1) or Pan-Heme (FusionPlex pan Heme EPOR add v1.2, Table S1). We required a minimum Average Unique RNA Start Sites per GSP2 Controls ≥ 10 and Read Depth Normalization ≥ 3,500,000 (for downsampling). Minimum passing quality control metrics per sample include: Q30 ≥70%, FASTQ Read Count ≥ 4,500,000 (if snap frozen tissue, peripheral blood, bone marrow, pleural fluid) or 8,000,000 (if FFPE tissue). The following metrics were only evaluated for snap frozen tissue, peripheral blood, bone marrow, and pleural fluid: On-Target Reads ≥ 80%, Average RNA Reads per GSP2 ≥ 90, Average Unique Start Sites per GSP2 ≥ 30, and GSP2s with greater than 20 Unique Start Sites ≥ 40%. FFPE tissues are only required to pass minimum thresholds for Q30 reads, FASTQ reads, Percent On-Target Reads, and Average Unique RNA Start Sites per GSP2 Controls. Reads were aligned to the human reference genome (GRCh37/hg19). Gene fusions and additional complex structural rearrangements (also referred to as isoforms), including ITDs and intragenic exon deletions, were ordered in a systematized schema using a laboratory-developed analysis workflow. Fusions and isoforms were prioritized for further interpretation if they were (1) gene-to-gene events and associated with strong evidence as described by the manufacturer; (2) gene-to-gene events, associated with weak evidence as described by the manufacturer, and deemed of high quality (i.e., is not classified as a mispriming event, known Ensembl paralog, transcript readthrough, containing intronic sequence, and out of reading frame); (3) intergenic event associated with genes described with immunoglobulin gene fusions and associated with strong evidence; or (4) intergenic event associated with immunoglobulin genes (*IGH*, *IGK*, *IGL*) and associated with weak evidence and deemed of high quality. Events previously reported were also prioritized for additional review. Additionally, we manually reviewed genes known to be associated with intragenic rearrangement (solid tumors: *BCOR*, *EGFR*, *FGFR1*, *MET*; hematological malignancy: *ERG*, *FLT3*, *IKZF1*, *PAX5*) within the software user interface for complex structural rearrangements, filtering for rarely observed novel and oncogenic isoforms noting exons skipped or exons out of order. Structural rearrangements were interpreted using the standard guidelines set forth by the Association for Molecular Pathology (AMP), American Society of Clinical Oncology (ASCO), and College of American Pathologists (CAP) [29].

### 2.5. Orthogonal Confirmation

Orthogonal confirmation of structural events was performed by multiple methodologies. Most frequently, structural rearrangements were confirmed by RT-PCR followed by bi-directional Sanger sequencing. For RT-PCR, 500 ng of RNA with MultiScribe reverse transcriptase (ThermoFisher, Waltham, MA, USA) and random hexamers (Applied Biosystems, Foster City, CA, USA) was used. PCR of the cDNA product was performed with primers designed to amplify across the breakpoint junction (primer sequences are available upon request). The PCR products were purified using the QIAquick purification kit (Qiagen, Germantown, MD, USA). Forward and reverse Sanger sequencing reactions were performed using the Big Dye v3.1 terminator mix (ThermoFisher, Waltham, MA, USA). Sequencing was performed on an Applied Biosystems 3730 or 3730XL instrument. For internal tandem duplications and selected gene fusions, FASTQ files from RNA sequencing generated from the Pan-Heme or Pan-Solid assays were run through the CICERO fusion calling software to confirm the reported event [26]. Additionally, in some cases, FISH was used for orthogonal confirmation.

### 2.6. Statistical Analysis

A chi-squared test or unpaired t-test was used to determine the significance between groups. *p* < 0.05 was considered significant.

## 3. Results

### 3.1. Overall Clinical Cohort Characteristics

Among the analyzed cohort, the average age at the time of testing was 25 years old (range: 0–82 years). Males underwent testing at a slightly higher frequency (57%) compared to females (43%). The Pan-Heme panel (*n =* 522, 86%) was ordered at a higher frequency compared to the Pan-Solid panel (*n =* 86, 14%).

### 3.2. Three-Year Diagnostic Yield for Hematologic Malignancies

**Figure 1:**
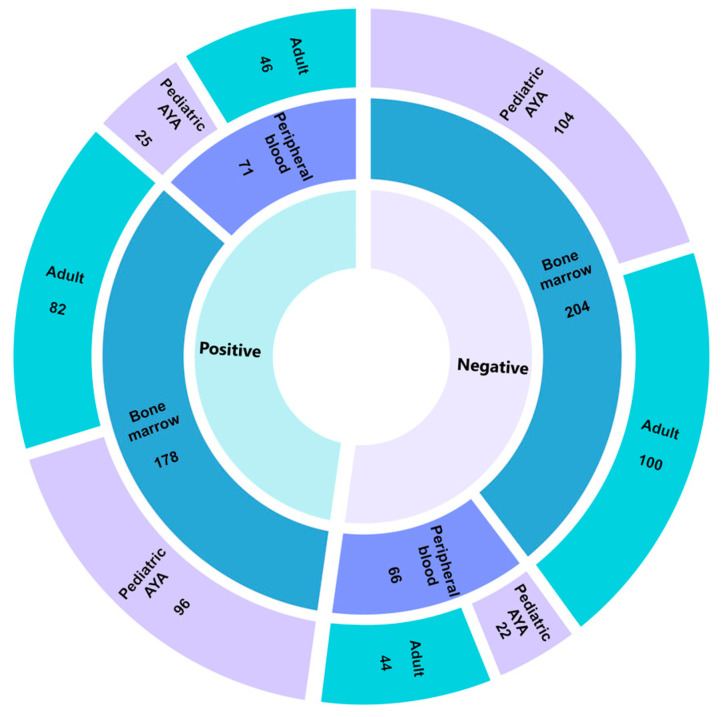
Pan-Heme diagnostic yield. Sunburst plot highlighting diagnostic yield across 522 individuals who underwent testing on the Pan-Heme panel. The innermost circle denotes positive (defined as either a Tier I or II event according to AMP/ASCO/CAP guidelines) or negative findings with tissue type and age group reported in the middle and outermost circles, respectively. AYA, adolescent/young adults.

We evaluated samples from 522 individuals with hematologic malignancies using an AMP-based targeted RNA sequencing hematologic panel (Table 2 and Table S2). The mean disease involvement (as measured in percentage of blasts) for the evaluated sample was 69% (median: 80%, range: 0–95%). A single patient with hypereosinophilic syndrome had an estimated disease involvement of 0%, as this diagnosis does not typically present with increased blasts. The mean age of testing was 28 years old (median: 20 years, range: 0–82 years). Overall, 382 (73%) and 137 (26%) samples were derived from disease-involved bone marrow and peripheral blood, respectively. Other tissue types analyzed included pleural fluid (*n =* 1), snap frozen lymph node tissue (*n =* 1), and FFPE lymph node tissue (*n =* 1). The diagnostic yield did not differ with regard to tissue type, with 51% of peripheral blood and 46% of bone marrow samples returning clinically actionable findings (*p* = 0.33, Figure 1). The indications for study were captured from the provided order requisition and predominantly included individuals with acute lymphoid leukemia, not otherwise specified (*n =* 222, 43%), and B-cell acute lymphoid leukemia (*n =* 226, 43%) (Table S2). Of the 522 total cases evaluated, 261 had a finding reported, with 251 of those classified as Tier I or Tier II for strong or potential clinical significance, respectively, according to the AMP/ASCO/CAP standard guidelines [29]. Findings reported as Tier III (variants of uncertain significance) were not considered positive for diagnostic yield calculations. Among gene fusions alone, we reported a diagnostic yield of 37% (183/522 cases). With the inclusion of intragenic structural rearrangements, we observed an increase in diagnostic yield to 48% (251/522 cases, Table S2). To further evaluate diagnostic yield across age groups, individuals were classified as ≤18 years old (pediatric adolescent/young adult (AYA)) and >18 years old (adults). No differences were appreciated in diagnostic yield between the pediatric/AYA (49%, 123/249 cases) and adult groups (47%, 128/273 cases) (*p* = 0.57) (Figure 1).

**Figure 2:**
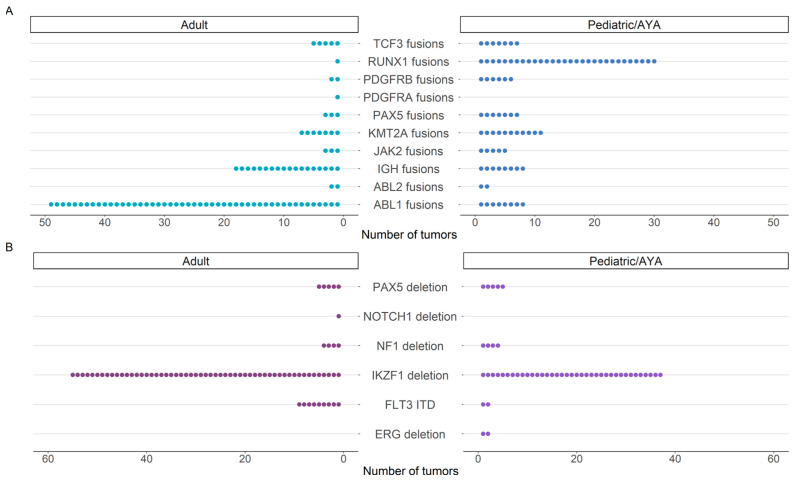
Frequency of events identified by the Pan-Heme assay. The number of (**A**) gene fusions and (**B**) intragenic structural rearrangements identified in individuals with hematologic malignancy according to age group.

Among clinically significant fusions in the adult cohort, *ABL1* and *ABL2* fusions were the most frequent, with *BCR::ABL1* fusion identified in 39/51 (76%) individuals (Figure 2). Of those 39 individuals with *BCR::ABL1* gene fusion, 37 (95%) presented with acute lymphoid leukemia. Among the pediatric/AYA cohort, the *ETV6::RUNX1* fusion typically associated with a favorable outcome in acute lymphoblastic leukemia was most frequent (Figure 2). Among both pediatric and adult cohorts, *IKZF1* deletion was the most common intragenic structural rearrangement, frequently involving deletion of exons 2-3, exons 2-6, exons 2-7, and exons 4-7 (Figure 2 and Figure S1). By expanding our analysis to include intragenic structural rearrangements, we identified that 10% (51/522) of individuals harbored an intragenic rearrangement concurrently with a gene fusion, with a statistically significant preference towards adults (12%, 34/273) relative to the pediatric/AYA (7%, 17/249) group (*p* = 0.03, Figure S1, Table S2). Additionally, we identified a single individual with B-cell precursor acute lymphoid leukemia with two intragenic deletions involving *IKZF1* and *PAX5*, in the absence of an *ERG* deletion, suggestive of the IKZF1^plus^ phenotype [30].

### 3.3. Three-Year Diagnostic Yield for CNS and Non-CNS Solid Tumors

**Figure 3:**
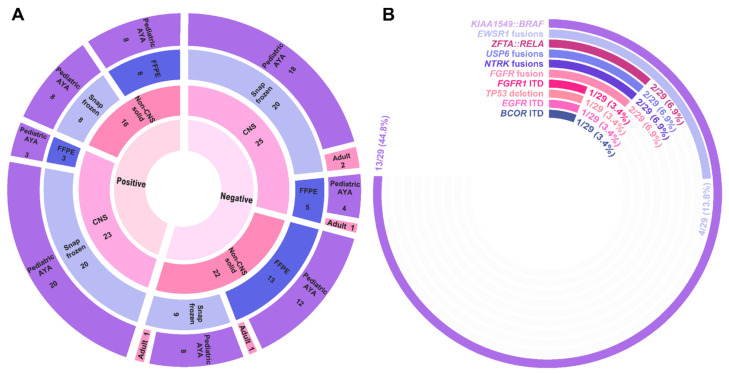
Pan-Solid diagnostic yield. (**A**) Sunburst plot highlighting diagnostic yield across 37 individuals who underwent testing on the Pan-Solid panel. The innermost circle denotes positive (defined as either a Tier I or II event according to AMP/ASCO/CAP guidelines) or negative findings with disease type, tissue type, and age group represented by the outer circles. CNS, central nervous system; FFPE, formalin-fixed paraffin embedded; AYA, adolescent/young adult. (**B**) Radial bar chart demonstrating the frequency of fusions and structural rearrangements identified by the Pan-Solid panel.

We evaluated 86 CNS and non-CNS solid tumors using an AMP-based targeted RNA sequencing solid tumor panel (Table 3 and Table S3). The mean disease involvement for the evaluated tumor sections was 78% (median: 85%, range: 10–100%). The mean age at testing was 9 years old (median: 8 years, range: 0–32 years), with a strong bias towards the pediatric population due to a predominant institutional patient population undergoing study. Only five individuals >18 years of age were evaluated by the Pan-Solid panel in this cohort. Fifty-seven (66%) samples were from snap frozen tissue, while the remaining 29 (34%) were derived from FFPE sections. This cohort included 38 (44%) individuals with non-CNS solid tumors and 48 (56%) individuals with CNS tumors. Of the 40 cases with a reportable finding, 39 (98%) were classified with strong or potential clinical significance (Tier I or Tier II) according to AMP/ASCO/CAP standard guidelines (Supplementary Table S3). Among reportable gene fusions alone, a diagnostic yield of 41% (35/86 cases) was observed. With the inclusion of intragenic structural rearrangements, the diagnostic yield increased to 45% (39/86 cases). CNS tumors (23/48 (48%)) and non-CNS tumors (16/38 (42%)) demonstrated a similar proportion of cases returning a clinically significant finding (*p* = 0.59, Figure 3A). Despite the differences in sequencing metrics between snap frozen and FFPE tissues, the diagnostic yield of these samples was comparable, with 38% of FFPE and 49% of snap frozen tumors returning a clinically significant finding (*p* = 0.32, Figure 3A).

Among detected fusions and isoforms, the majority (88%) represented events previously described in the literature and/or databases on the basis of gene partners (Table S3). The well-described *KIAA1549::BRAF* fusion was most frequently observed, being identified in 13/23 (57%) CNS tumors with a clinically significant finding (Figure 3B). While the majority of clinically significant events were gene fusions (*n =* 33), three cases harbored a complex structural rearrangement resulting in tandem duplication, including *EGFR* ITD (Solid_19), *FGFR1* ITD (Solid_86), and *BCOR* ITD (Solid_68). In total, 36/40 (90%) cases harbored a single reportable finding. Four cases harbored multiple isoforms of the reportable gene fusion event: *MYH9::USP6* (Solid_35), *SOX10::NTRK3* (Solid_69), *SQSTM1::NTRK2* (Solid_64), and *CHCHD7::PLAG1* (Solid_21) (Table S3). Additionally, Solid_21 also harbored a second, independent PLAG1 fusion (*ZFHX4::PLAG1*). 

### 3.4. Clinical Utility of Identified Events

We evaluated the clinical utility of the identified genomic events as related to the tumor type provided for testing. Events reported as Tier I or Tier II according to the AMP/ASCO/CAP guidelines were evaluated for clinical utility. In total, 217 of 608 (36%) patient samples harbored a gene fusion event classified as Tier I or Tier II providing meaningful information to inform diagnosis. In relation to the observed genomic event, our testing provided prognostic information for 90% (*n =* 196) of cases and demonstrated potential for change to therapeutic management in 64% (*n =* 138) of cases (Table S4). In total, 127 cases were identified with an intragenic deletion or ITD. Of those, there were nine unique, non-redundant events identified, including ITDs of the *BCOR* (*n =* 1), *EGFR* (*n =* 1), *FGFR1* (*n =* 1), and *FLT3* (*n =* 11) genes, and intragenic deletions of *ERG* (*n =* 2), *IKZF1* (*n =* 92), *NF1* (*n =* 8), *NOTCH1* (*n =* 1), and *PAX5* (*n =* 10) genes. These intragenic events provided information relating to diagnostic refinement (7/9 (78%) events), prognostication (3/9 (33%) events), and/or identification of potential targeted therapy (3/9 (33%) events) (Table S5).

The clinical utility and implications of this testing have been reported on five individuals within this cohort. We reported on an individual with an epithelioid tumor of the omentum which was found to harbor a *PRRC2B::ALK* fusion (Solid_13). Based on the presence of an *ALK* rearrangement (initially identified by FISH and refined by AMP-based RNA sequencing), the individual was offered targeted therapy with the ALK inhibitor, crizotinib [31]. We also presented a 2-year-old with a congenital inguinal mass [32]. Through AMP-based RNA sequencing, we identified two different isoforms of a *CHCHD7::PLAG1* fusion and a *ZFHX4::PLAG1* fusion (Solid_21). These fusion events are predicted to result in promoter swapping, whereby the 5′ gene partners *CHCHD7* or *ZFHX4* may drive expression of full-length *PLAG1*. *PLAG1*-rearranged tumors may present with multiple 5′ fusion partners, and emerging data suggest that the histologic spectrum of this entity is expanding [33,34,35]. Furthermore, we also previously reported on an infant with a congenital midline lesion associated with a closed spinal dysraphism [36]. In this atypical lesion, we identified an *FGFR1* ITD (Solid_86) which is exceedingly rare outside of CNS tumors, highlighting the expanding phenotypic spectrum to include benign lesions. Additionally, we reported on an infant who presented with a renal mass for which an *EGFR* ITD (Solid_19) was identified [37]. The presence of the *EGFR* ITD confirmed a diagnosis of congenital mesoblastic nephroma. Lastly, we described a 2-year-old female with a *CIC::NUTM1* sarcoma (Solid_47) and highlighted the histological overlap with primitive myxoid mesenchymal tumor of infancy [38]. 

## 4. Discussion

Clinical testing to identify a wide breadth of gene fusion or complex structural rearrangement events across many genes and breakpoints is becoming increasingly critical for guiding diagnostic, prognostic, and therapeutic information. The capabilities of NGS-based assays to simultaneously evaluate gene fusions and complex structural rearrangements has the potential to improve patient care by reducing time to molecular diagnosis and improving diagnostic yield. Notably, the 2021 World Health Organization (WHO) Classification of Tumours of the Central Nervous System transformed the reporting schema for CNS tumor diagnostics, promoting the integration of molecular and histopathological data to delineate a final integrated diagnosis [39]. Herein, we describe our three-year experience using AMP-based targeted RNA sequencing among pediatric and adult populations and the expansion of our reporting paradigm to include both gene fusions and complex structural rearrangements. This study highlights the capability and utility of AMP-based targeted RNA fusion panels as a diagnostic tool for reporting clinically significant complex structural rearrangements, in addition to gene fusions.

Multiple groups have demonstrated the feasibility and utility of AMP-based targeted RNA sequencing panels to identify gene fusions across a breadth of hematologic, CNS, and non-CNS solid tumors in the diagnostic setting [40,41,42,43]. Panel composition across studies varied from 53 to 112 genes, with enrichment for targets in both solid tumors (*n =* 4 studies) and hematologic malignancies (*n =* 1 study). Across these studies, the reported diagnostic yield was broad (3.8–61%), owing to differences in patient populations and tumor types under evaluation [40,41,42,43]. Notably, complex structural rearrangements were not well represented among these prior studies, with only a single group reporting the well-described *MET* and *EGFR* exon skipping events [42]. Expanding on these prior studies, we evaluated diagnostic yield across a wide breadth of hematologic malignancies and solid tumors, reporting clinically significant complex structural rearrangements in addition to gene fusions. Similar to prior studies, the diagnostic yield of our assay was 36% (37% hematologic malignancies, 41% solid tumors) when considering only gene fusions. By including a report of complex structural rearrangements, we increased our combined diagnostic yield to 48% (48% hematologic malignancies, 45% solid tumors). Thus, we provide evidence demonstrating the clinical utility of AMP-based targeted RNA sequencing methodologies for the identification of complex structural rearrangements. 

The identification of complex structural rearrangement may have clinical significance for individuals with hematologic and solid malignancies. For example, *IKZF1* encodes for IKAROS, a regulator of lymphoid differentiation. The *IKZF1* deletion was the most common event observed amongst both pediatric/AYA and adult Pan-Heme cohorts (Figure 2). Deletion of *IKZF1*, most frequently whole gene deletion, is found in 25–35% of adults and 12–17% of children with acute lymphoid leukemia and confers a poorer prognosis [11,21,44]. A high correlation between the *BCR::ABL1* fusion and *IKZF1* deletion has been reported in individuals with B-cell acute lymphoid leukemia, with both events serving as independent risk factors associated with poor prognosis [45]. *PAX5* intragenic deletions were also frequently identified in our hematologic cohort (Figure 2). *PAX5* encodes paired box 5, a transcription factor essential for regulating the B cell lineage differentiation [46]. Previously described intragenic deletions have been shown to abolish the homeodomain-like domain and part of the transactivation domain, resulting in a loss of function [47,48,49]. PAX5 loss arrests B cell differentiation at immature precursor stages to promote leukemogenesis [46]. *FLT3* was the most frequently observed ITD among the Pan-Heme cohort. *FLT3* encodes a receptor tyrosine kinase that is widely expressed among hematopoietic progenitor cells [50]. *FLT3* ITD is observed in about 30% of acute myeloid leukemia and is associated with poor risk [12,50,51]. FLT3 inhibitors have demonstrated clinical efficacy among this patient population, although resistance remains a challenge [4]. While intragenic structural rearrangements were not as frequent among our Pan-Solid cohort, we identified three cases harboring an ITD in our solid tumor cohort. In the setting of a CNS tumor, the presence of a *BCOR* ITD may provide diagnostic information, defining a molecular entity in the WHO Classification of CNS Tumours known as CNS Tumour with *BCOR* ITD [39,52,53,54,55,56,57]. In the kidney, the identification of a *BCOR* ITD is diagnostic for clear cell sarcoma of the kidney [57,58,59]. In the soft tissue, *BCOR* genetic alterations, including *BCOR* ITD, are diagnostically defining for a sarcoma that includes undifferentiated round cell sarcoma and primitive myxoid mesenchymal tumor of infancy morphologic types [60]. Similarly, *EGFR* ITDs are described in the setting of infantile fibrosarcoma and congenital mesoblastic nephroma, particularly in classic or mixed subtype, and aids in diagnostics if the tumor is negative for the more commonly identified *ETV6::NTRK3* fusion. This scenario further highlights the utility of broader panel testing and analysis to encompass recurrent events to enable alignment for an integrated diagnosis along with histopathologic characteristics [19,20,37,61]. *FGFR1* ITDs are commonly reported in the setting of low-grade glial tumors for which the therapeutic significance is still under study [5,8,15].

There are technical aspects of this multiplexed targeted RNA panel that offer improvements over the current methodologies. For example, FISH utilizes fluorescent probes targeted to regions of interest for a particular gene or locus. FISH is a rapid and cost-effective method to identify regions of genomic loss or gain or structural rearrangement; however, it is highly targeted and cannot accurately determine the downstream impact on transcripts or proteins. If a tumor harbors an atypical fusion partner or breakpoint, a very focal event, or a complex event, FISH may be unable to detect it. Multiplexed NGS assays have demonstrated cost-benefit and reduced turnaround time compared to single gene testing in a variety of cancer types [62,63,64]. AMP-based chemistry allows for the identification of both recurrent fusions and structural rearrangements and importantly those that are novel or may harbor atypical breakpoints [65]. The ability to fully characterize a structural rearrangement can have clinical impact. For example, the *EWSR1* gene generates fusion transcripts with multiple gene partners. While break-apart *EWSR1* FISH probes are frequently used for diagnostic testing to determine if an *EWSR1* rearrangement is present, this assay is not able to identify the fusion partner. The 3′ gene partner is important for diagnostic classification as various partners (e.g., *FLI1* in Ewing sarcoma, *WT1* in desmoplastic small round cell tumor) are characteristic of distinct tumor entities [66]. While some ITDs have recurrent breakpoints, the majority are not recurrent, may be different sizes, and can include unaligned or intronic linker sequence. Notably, we have demonstrated that AMP chemistry can call intragenic structural rearrangements across a wide breadth of sizes, with an average size for *FLT3* ITD (*n =* 11) of 64 bp (range: 21–198 bp) (Table S2). Intragenic deletions for hematologic malignancies (*n =* 113) typically spanned multiple exons, with an average size of 108,545 bp (range: 41,536–139,851 bp). While ITDs were less commonly seen amongst solid tumors (*n =* 3), the size tended to be larger than what was observed for *FLT3* ITD (Table S3), with a *BCOR* ITD of 90 bp and an *EGFR* ITD of 27,434 bp representing the smallest and largest events in this cohort, respectively.

This assay also demonstrates technical limitations that should be considered when evaluating and clinically interpreting intragenic, complex structural rearrangements. In tumors for which a *FLT3* ITD is identified, the frequency or allelic ratio is necessary to fully delineate the prognostic implications of this event [51,67]. Our implementation of AMP-based targeted RNA sequencing does not enable a determination of the allelic ratio. Additionally, the ability of this assay to detect very small ITD events is expected to be limited. Furthermore, our assay was not designed to detect whole gene (or larger) gains or losses in the form of copy number variation. Gene-level and larger *IKZF1* deletions associated with haploinsufficiency would not be reportable through this assay [11,68]. Moreover, the bioinformatic identification of complex structural rearrangements using the manufacturer’s analysis software user interface may pose challenges if the event is not curated within the Quiver database (http://quiver.archerdx.com/ accessed on 31 July 2023). Curated events, such as the *EGFR* ITD and *IKZF1* deletion, are classified as oncogenic isoforms within the manufacturer’s analysis software and thus prioritized for analysis. Many of the newly described clinically relevant intragenic deletions and ITDs (e.g., *FGFR1* ITDs) are not curated. Therefore, identifying these events typically requires manual review amongst many false positive fusions and isoforms and readthrough events. Additionally, the reporting of small intraexon insertions/deletions (termed “intraexon gap” in the Archer UI) which have not been curated within the Quiver database (i.e., *FLT3* ITD and *BCOR* ITD) remains a challenge as fragmented nucleic acids, such as those from FFPE samples, tend to have an elevated level of false positive intraexon gap events based on our experience. While calling these events is technically possible with this methodology, it will require significant validation to ensure accurate reporting. The incorporation of other bioinformatic tools, such as CICERO [26], may assist in identifying complex structural rearrangements, particularly ITDs, and improve the ability to distinguish artifacts from true, medically meaningful events. While our laboratory testing scheme currently incorporates orthogonal confirmation for many reportable events, the use of orthogonal confirmation may vary between laboratories.

There are limitations to consider for this retrospective study. While our institution serves a pediatric population, we also serve as a reference lab for the testing of both pediatric and adult samples, as evident in our Pan-Heme cohort, where 52% of cases were >18 years old. However, our Pan-Solid cohort consisted predominantly of a pediatric population (94% of individuals ≤18 years old at the time of testing) derived predominantly from in-house testing. The inherent differences in the etiology of pediatric and adult tumors should be considered when interpreting these data as pediatric tumors are more likely to have a low somatic mutation burden and be driven by gene fusions or structural rearrangements [69,70]. The genetic landscape for pediatric and adult tumors is constantly evolving and ensuring gene content for panel-based testing relevant to the patient population under study is critical. Both Pan-Heme and Pan-Solid panels used in our laboratory were customized to include additional gene specific primers targeting genes associated with pediatric cancers.

## 5. Conclusions

In summary, we describe the three-year diagnostic yield of clinical targeted RNA sequencing using AMP-based library preparation. The cohort tested at our institution represents a wide spectrum of demographics, including pediatric/AYA and adult cohorts evaluated for hematologic and solid cancers, using diverse tissue types, including FFPE, snap frozen, peripheral blood, and bone marrow. We demonstrate an increase in diagnostic yield when complex structural rearrangements, including intragenic deletions and ITDs, are reported by this methodology, expanding on the provision of clinically useful information as related to diagnostic classification, prognostication, and therapeutic determination in this cohort. 

## Figures and Tables

**Table 1:** Comparison of methodology.

	Conventional Karyotype	Fluorescence In Situ Hybridization	Conventional Microarray	RT-PCR	Genome Sequencing	AMP-Based RNA-Seq (e.g., Archer)	RNA-Seq
Analyte	Metaphase Chromosomes	Interphase nuclei or Metaphase Chromosomes	DNA	RNA	DNA	RNA	RNA
Estimated turnaround time ^¥^	3–7 days	1–2 days	3–7 days	1–5 days	7–14 days	7–14 days	7–14 days
Targeted	No	Yes	No	Yes	No	Yes	No
Gene fusion resolution	Cytogenetic Resolution (Mb); Breakpoints hone and refine likely involved genes	Gene locus resolution (Mb- kb); Probe signal patterns hone and refine involved genes	Molecular Resolution (kb- bp); Breakpoints hone and refine involved genes	Molecular Resolution (bp); Confirms both gene partners	Molecular Resolution (bp); Confirms both gene partners	Molecular Resolution (bp); Confirms both gene partners	Molecular Resolution (bp); Confirms both gene partners
Intragenic deletion *	No	No	Yes	Yes	Yes	Yes	Yes
ITD resolution *	No	No	Yes	Yes	Yes	Yes	Yes

ITD: internal tandem duplication, Mb: Megabase, kb: kilobase, bp: basepair, ^¥^ Turnaround times are estimated and are laboratory-dependent, * Identification of intragenic deletion or ITD may be size-dependent.

**Table 2:** Demographics for individuals undergoing testing on the Pan-Heme assay.

	Individuals (*n =* 522)
**Sex**	
Male	306 (59%)
Female	216 (41%)
**Age at the time of testing**	
0–18 years	249 (48%)
>18 years	273 (52%)
**Specimen source**	
Bone marrow	382 (73%)
Peripheral blood	137 (26%)
Other	3 (1%)
**Indication for testing**	
Acute lymphoid leukemia	222 (43%)
B-cell acute lymphoid leukemia	226 (43%)
Acute leukemia, NOS	33 (6%)
Acute myeloid leukemia	5 (1%)
MPO negative blasts	4 (1%)
Acute megakaryoblastic leukemia	3 (1%)
Abnormal B cell population	3 (1%)
Chronic myeloid leukemia	2 (<1%)
Diffuse large B cell lymphoma	2 (<1%)
Mixed lineage leukemia	2 (<1%)
Myeloid neoplasm	2 (<1%)
Eosinophilia	1 (<1%)
Myelodysplastic syndrome	1 (<1%)
Other	11 (2%)
Indication not provided	5 (1%)
Myeloid neoplasm	2 (<1%)

Tissue type—other includes pleural fluid and lymph node. Indication for testing—others include elevated white blood count, hypereosinophilic syndrome, lymphoma, pancytopenia, ring chromosome 7, thrombocytopenia, thrombocytosis, and concern for hematologic disease without diagnosis. NOS, not otherwise specified; MPO, myeloperoxidase.

**Table 3:** Demographics for individuals undergoing testing on the Pan-Solid assay.

	Individuals (*n =* 37)
**Sex**	
Male	40 (47%)
Female	26 (53%)
**Age at the time of testing**	
0–18 years	81 (94%)
>18 years	5 (6%)
**Specimen source**	
Snap frozen	57 (66%)
Formalin-fixed paraffin-embedded	29 (34%)
**Tumor type**	
Non-CNS solid tumor	38 (44%)
CNS tumor	48 (56%)

CNS: central nervous system.

## Data Availability

The data that support the findings of this study are available on request from the corresponding author. The data are not publicly available due to privacy or ethical restrictions.

## Supplementary Materials

The following supporting information can be downloaded at: https://www.mdpi.com/article/10.3390/cancers15174394/s1, Figure S1: Co-occurrence of gene fusions and intragenic structural rearrangements, Table S1: Pan-Heme and Pan-Solid gene lists, Table S2: Pan-Heme Gene Fusions and Structural Rearrangements, Table S3: Pan-Solid Gene Fusions and Structural Rearrangements, Table S4: Gene Fusion Clinical Outcomes, Table S5: Intragenic Structural Rearrangement Clinical Outcomes.
