# Supplementary figures and images for: Expanding the Clinical Utility of Targeted RNA Sequencing Panels beyond Gene Fusions to Complex, Intragenic Structural Rearrangements
Source: Cancers (Basel). 2023 Sep 2;15(17):4394. doi: 10.3390/cancers15174394 (PMC10486946; doi:10.3390/cancers15174394)

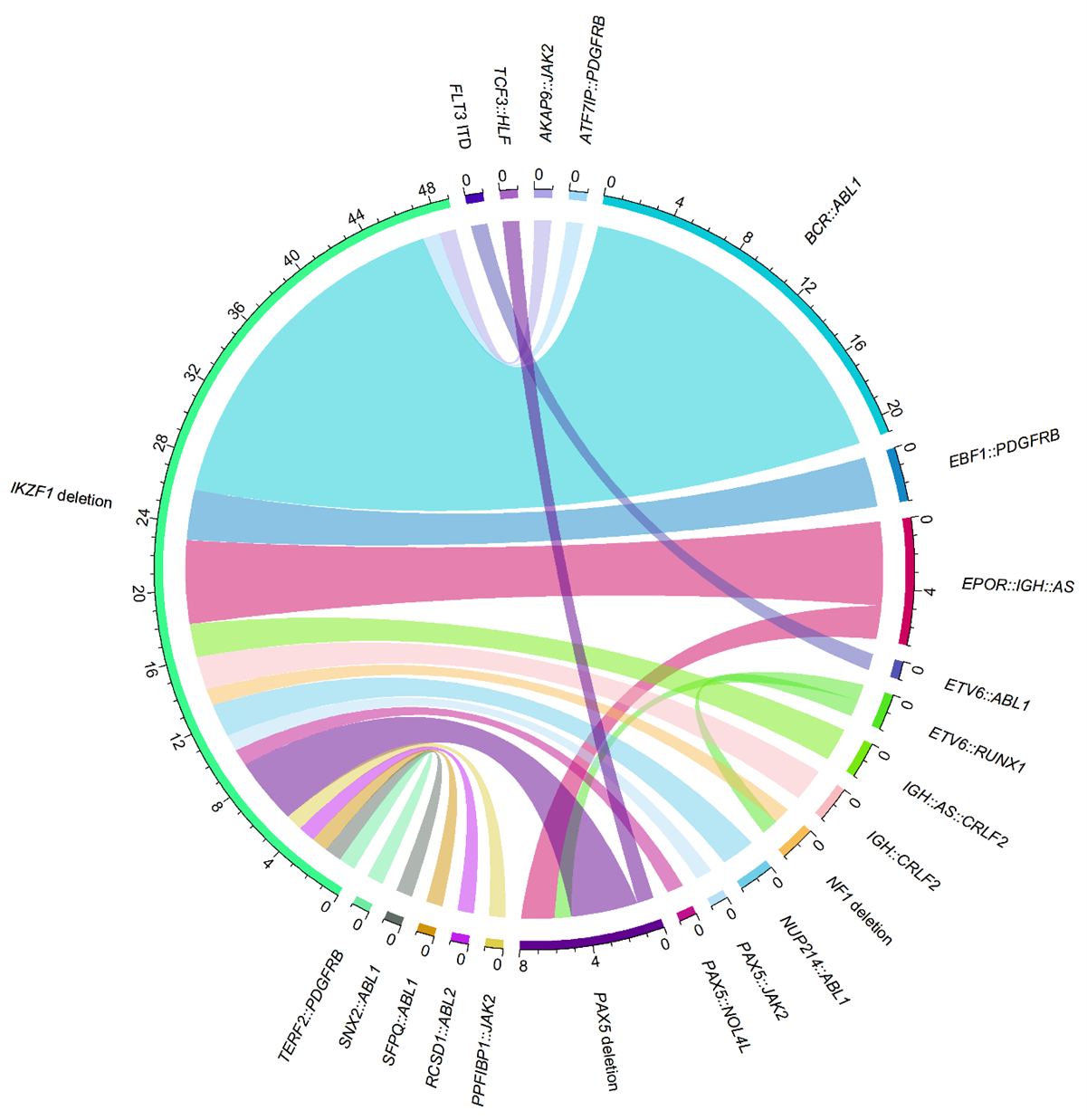

Supplement: Supplementary file 1 [file cancers-15-04394-s001.zip › Figure S1.png]
